# Chemokine CCL2 and its receptor CCR2 in the medullary dorsal horn are involved in trigeminal neuropathic pain

**DOI:** 10.1186/1742-2094-9-136

**Published:** 2012-07-09

**Authors:** Zhi-Jun Zhang, Yu-Lin Dong, Ying Lu, Su Cao, Zhi-Qi Zhao, Yong-Jing Gao

**Affiliations:** 1 Institute of Nautical Medicine, Jiangsu Key laboratory of Neuroregeneration, Nantong University, 19 Qixiu Road, Nantong, 226001, China; 2 Department of Anatomy, Medical School of Nantong University, Nantong, 226001, China; 3 Institute of Neurobiology, Institutes of Brain Science and State Key Laboratory of Medical Neurobiology, Fudan University, Shanghai, 200032, China

## Abstract

**Background:**

Neuropathic pain in the trigeminal system is frequently observed in clinic, but the mechanisms involved are largely unknown. In addition, the function of immune cells and related chemicals in the mechanism of pain has been recognized, whereas few studies have addressed the potential role of chemokines in the trigeminal system in chronic pain. The present study was undertaken to test the hypothesis that chemokine C-C motif ligand 2 (CCL2)-chemokine C-C motif receptor 2 (CCR2) signaling in the trigeminal nucleus is involved in the maintenance of trigeminal neuropathic pain.

**Methods:**

The inferior alveolar nerve and mental nerve transection (IAMNT) was used to induce trigeminal neuropathic pain. The expression of ATF3, CCL2, glial fibrillary acidic protein (GFAP), and CCR2 were detected by immunofluorescence histochemical staining and western blot. The cellular localization of CCL2 and CCR2 were examined by immunofluorescence double staining. The effect of a selective CCR2 antagonist, RS504393 on pain hypersensitivity was checked by behavioral testing.

**Results:**

IAMNT induced persistent (>21 days) heat hyperalgesia of the orofacial region and ATF3 expression in the mandibular division of the trigeminal ganglion. Meanwhile, CCL2 expression was increased in the medullary dorsal horn (MDH) from 3 days to 21 days after IAMNT. The induced CCL2 was colocalized with astroglial marker GFAP, but not with neuronal marker NeuN or microglial marker OX-42. Astrocytes activation was also found in the MDH and it started at 3 days, peaked at 10 days and maintained at 21 days after IAMNT. In addition, CCR2 was upregulated by IAMNT in the ipsilateral medulla and lasted for more than 21 days. CCR2 was mainly colocalized with NeuN and few cells were colocalized with GFAP. Finally, intracisternal injection of CCR2 antagonist, RS504393 (1, 10 μg) significantly attenuated IAMNT-induced heat hyperalgesia.

**Conclusion:**

The data suggest that CCL2-CCR2 signaling may be involved in the maintenance of orofacial neuropathic pain via astroglial–neuronal interaction. Targeting CCL2-CCR2 signaling may be a potentially important new treatment strategy for trigeminal neuralgia.

## Background

Neuropathic pain resulting from many types of injury to the nervous system is a devastating disease. The mechanisms by which nerve injury develops neuropathic pain have remained largely unknown. It was generally believed that only neurons and their neural circuits were responsible for the development and maintenance of neuropathic pain. In recent years, it is increasingly recognized that non-neuronal cells such as immune cells and glial cells also play a critical role in the pathogenesis of neuropathic pain [1-5]. Both astrocytes and microglia were activated in the spinal cord [6-8] and trigeminal nucleus [9-11] following peripheral nerve injuries such as nerve transection and ligation. The activated glial cells can contribute to the enhancement and maintenance of neuropathic pain by releasing potent neuromodulators, such as growth factors, proinflammatory cytokines and chemokines [12-16]. In particular, chemokines have been demonstrated to be involved in neuroinflammation at different anatomical locations, including injured nerve, dorsal root ganglion (DRG), spinal cord, and brain [17-20] and contribute to chronic pain processing [16].

Chemokine C-C motif ligand 2 (CCL2), also known as monocyte chemoattractant protein 1 (MCP-1), is a member of the chemokines family and can specifically recruit monocytes to sites of inflammation, infection, trauma, toxin exposure, and ischemia. The biological effects of CCL2 are mediated via interaction with its G protein-coupled receptor, chemokines C-C motif receptor 2 (CCR2) [21]. Targets of CCR2 signaling include mitogen-activated protein kinase (MAPK) [22], an important intracellular signaling in regulating neural plasticity and inflammatory responses [23], indicating CCL2-CCR2 may be involved in neuroinflammation and chronic pain. Indeed, behavioral studies have shown that mice lacking CCR2 display a substantial reduction in mechanical allodynia after partial ligation of the sciatic nerve [24,25]. Mice overexpressing CCL2 in astrocytes exhibit enhanced pain sensitivity [26]. Although CCL2 and CCR2 expression are well documented in the DRG in conditions of nerve injury [27-30] and tissue inflammation [31], the expression of CCL2 and CCR2 in the spinal cord is debated [32,33]. In addition, whether CCL2-CCR2 signaling is involved in trigeminal neuropathic pain remains unknown.

The trigeminal spinal subnucleus caudalis, which has a laminated structure similar to the spinal dorsal horn and is often referred as the medullary dorsal horn (MDH), has been generally considered to play an essential role in trigeminal pain transmission [34,35]. Several animal models, such as injuries to the lingual nerve, infraorbital nerve (ION), inferior alveolar nerve (IAN), or the inferior alveolar nerve and mental nerve (IAMN) have been used to study the trigeminal neuropathic pain [9,10,36-40]. In addition, it was shown that after transection of IAN or IAMN, the whisker pad area, which is innervated by the ION, showed hypersensitivity to mechanical stimulation [9,40], suggesting the secondary hyperalgesia was induced. In the present study, we transected the IAMN, tested heat hyperalgesia in the whisker pad area, and investigated the expression and cellular distribution of CCL2 and CCR2 in the MDH after IAMN transection (IAMNT) in mice. The analgesic effect of CCR2 antagonist, RS504393 on IAMNT-induced heat hyperalgesia was also investigated at day 3 and day 10 after IAMNT.

## Methods

### Animals and surgery

**Figure 1:**
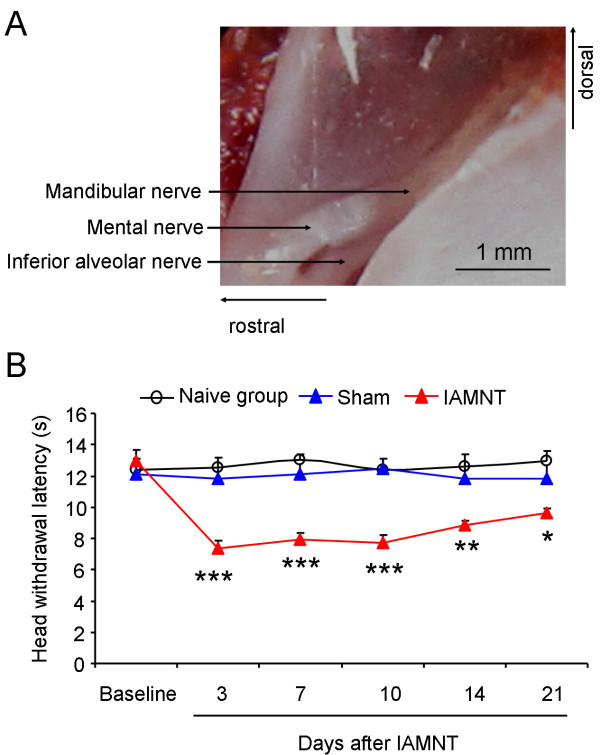
**Image showing the anatomical structure of the MN and the IAN and heat hyperalgesia produced after IAMNT. (A)** after removing the mandibular bone, the mandibular nerve, MN and IAN were exposed. Transection was made on the MN and IAN. **(B)** IAMNT induced significant decrease of head-withdrawal latency (HWL) on the ipsilateral facial skin. There were no significant changes of HWL in naïve and sham-operated mice. * *P <* 0.05, ** *P <* 0.01, *** *P <* 0.001, compared to sham-operated mice. *n =* 6 for each group.

Adult CD1 mice (male, 25–30 g) were purchased from the Experimental Animal Center of Nanjing Medical University. Mice were housed in plastic cages, and maintained on a 12:12 hour light/dark circle under conditions of 23 ± 1 °C with food and water available. All surgical and experimental procedures were reviewed and approved by the Animal Use and Care Committee for Research and Education of Nantong University. Animal treatments were performed according to the Guidelines of the International Association for the Study of Pain [41]. Before the experiments, the animals were allowed to habituate to the housing facility for 3 days. The IAN and mental nerve (MN) transection were made as described by Piao *et al*. [9]. In brief, mice were anesthetized with sodium pentobarbital (40–50 mg/kg). The facial skin over the left masseter muscle was cut and the mandibular bone was exposed. The bone surface was carefully removed and both the IAN and MN were exposed and transected (Figure 1A). Then the cutaneous tissue was sutured. For sham-operated mice, the facial skin and the masseter muscle were similarly incised and the IAN/MN was exposed, but no nerve transection was carried out.

### Drugs and administration

The selective CCR2 antagonist, RS504393 was purchased from Tocris (Bristol, UK). For intracisternal injection, animals were anesthetized with isoflurane. The medulla puncture was made with a 30 G needle between the occipital bone and the atlas to deliver the reagents (10 μl) to the cerebral spinal fluid.

### Behavioral experiments

Animals were habituated to the testing environment daily for at least two days before baseline testing. The room temperature and humidity remained stable for all experiments. Animals were put in plastic boxes and allowed 30 minutes for habituation before examination. A heat stimulus was applied to the maxillary whisker pad skin by radiant heat (IITC Life Science, Woodland Hills, CA, USA). Heat sensitivity was expressed as head-withdrawal latency (HWL). The radiant heat intensity was adjusted so that basal HWL is between 10–13 seconds, with a cut-off of 18 seconds to prevent tissue damage.

### Immunohistochemistry

After appropriate survival times, animals were deeply anesthetized with isoflurane and perfused through the ascending aorta with phosphate-buffered saline (PBS) followed by 4% paraformaldehyde in 0.1 M PB. After the perfusion, the medulla and upper cervical cord and trigeminal ganglion (TG) were removed and postfixed in the same fixative overnight. The medulla sections (30 μm, free-floating) and TG sections (14 μm) were cut in a cryostat and processed for immunofluorescence staining. The sections were first blocked with 2% goat or donkey serum for 1 hour at room temperature and then incubated overnight at 4 °C with the following primary antibodies: ATF3 antibody (rabbit, 1:1000, Santa Cruz, Santa Cruz, CA, USA), CCL-2 antibody (rabbit, 1:500, Millipore, Billerica, MA, USA), glial fibrillary acidic protein (GFAP) antibody (mouse, 1:5000, Millipore), OX-42 antibody (mouse, 1:5000, Serotec, Kidlington, Orford, UK), NeuN antibody (mouse, 1:5000, Millipore), Iba-1 antibody (rabbit, 1:5000, Wako, Tokyo, Japan), and CCR2 antibody (goat, 1:50, Santa Cruz). The sections were then incubated for 1 hour at room temperature with Cy3- or FITC-conjugated secondary antibodies (1:400, Jackson ImmunoResearch, West Grove, PA, USA). For double immunofluorescence, sections were incubated with a mixture of mouse and rabbit (or goat) primary antibodies followed by a mixture of FITC- and CY3-conjugated secondary antibodies. The stained sections were examined with a Leica fluorescence microscope, and images were captured with a CCD Spot camera. The sections with double staining were imaged with an Olympus FV10i confocal microscope.

### FG retrograde labeling

Naïve or IAMNT-operated (1 day) mice were anesthetized with isoflurane and 4 μl of 2% FG dye was subcutaneously injected into the mental skin. Two days later, the animals were perfused and the TGs were dissected and cut as described above.

### Western blot

After appropriate survival times, animals were transcardially perfused with 0.01 M PBS. The medulla was dissected. The tissues were homogenized in a lysis buffer containing protease and phosphatase inhibitors (Sigma, St Louis, MO, USA). Protein concentrations were determined by BCA Protein Assay (Pierce, Rockford, IL, USA). Thirty μg of proteins were loaded for each lane and separated on SDS-PAGE gel. After the transfer, the blots were incubated overnight at 4 °C with polyclonal antibody against CCR2 (goat, 1:100, Santa Cruz). For loading control, the blots were probed with glyceraldehyde-3-phosphate dehydrogenase (GAPDH) antibody (mouse, 1:20000, Millipore). These blots were further incubated with horseradish peroxidase-conjugated secondary antibody, developed in enhanced chemiluminescence solution, and exposed on Hyperfilm (Bio-Rad, Hercules, CA, USA) for 1–5 minutes. Specific bands were evaluated by apparent molecular size. The intensity of the selected bands was analyzed using Image J software (NIH, Bethesda, MD, USA).

### Rotarod test

Mice were trained to maintain their position on a rotarod (diameter, 3 cm) at 8 rpm for 60 seconds using the rotarod apparatus (Ugo Basile, Varese, Italy), and training was considered complete when mice were able to remain on the rotarod for 60 seconds. After intracisternal injection of RS504393, each mouse was placed back on the rotarod and latency to fall was recorded. The cut-off was set at 60 seconds.

### Quantification and statistics

The percentage of FG-labeled cells and ATF3-immunoreactive cells in the TG was quantified in 5 TG sections of each mouse (3 mice for each group). For the analysis of CCL2- or GFAP-immunoreactivity, the images of the brain stem sections from the level of 300 μm rostral to the obex to 1800 μm caudal to the obex were captured, the dorsomedial quarter of each section was outlined and a numerical value of the intensity was calculated with a computer-assisted imaging analysis system (Image J, NIH). The intensity of the background was subtracted in each section. The data from 5–6 animals in each group was averaged and analyzed. For western blot, the density of specific bands was measured with Image J. All data were expressed as mean ± SEM. For immunostaining and western blot studies, differences between two groups (Naïve vs. IAMNT) were compared using Student *t*-test. For behavioral studies, the data were analyzed with two-way ANOVA with repeated measurement followed by a Bonferroni’s *post hoc* tests. The criterion for statistical significance was *P <* 0.05.

## Results

### Time course of IAMNT-induced heat hyperalgesia

After two days habituation, the basal HWL of the whisker pad to heat stimulus was tested for naïve, IAMNT and sham-operated mice and they showed similar latencies (Naïve, 12.4 ± 0.7 seconds; IAMNT, 12.9 ± 0.8 seconds; Sham, 12.1 ± 0.7 seconds). The sham-operated and naïve groups did not show any significant changes at all time points, and no significant difference was observed between the sham and the naïve groups (*P* > 0.05). However, IAMNT induced significant decrease of HWL of the ipsilateral whisker pad compared with the sham group (*P <* 0.001). HWL decrease appeared on day 3 (7.4 ± 0.5 seconds) and maintained for more than 21 days (9.6 ± 0.3 seconds) after surgery (Figure 1B). These results indicate that IAMNT induces persistent trigeminal neuropathic pain.

### Decrease of FG-labeled neurons and increase of ATF3-IR neurons in the TG after IAMNT

**Figure 2:**
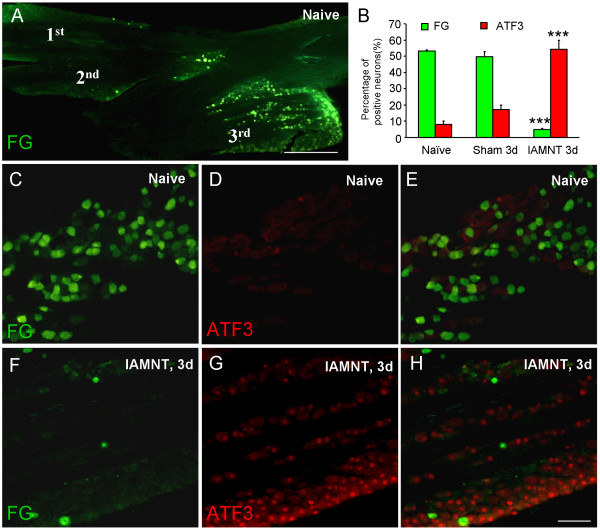
**The distribution of FG labeled neurons and expression of ATF3 in the TG in naïve, sham-operated, and IAMNT mice.** FG-labeled neurons were mainly distributed in the mandibular division (3rd) of TG in naïve mice **(A-C)** and decreased after IAMNT **(B,F)**. The ATF3 expression was increased after IAMNT **(B,D,G)**. No obvious colocalization of FG and ATF3 were found **(E,H)**. *** *P <* 0.001 (vs naïve). Scale bars, 500 μm in A, 100 μm in C–H. *n =* 3 for each group.

Two days after FG subcutaneous injection into the mental skin in naïve mice, many FG-labeled neurons (53 ± 0.8%) were observed in the mandibular division of TG (Figure 2A–C). However, few of the FG-labeled neurons were seen in the ophthalmic and maxillary divisions. In IAMNT-operated mice, FG-labeled neurons were dramatically decreased (4.9 ± 0.7%, *P <* 0.001) in the mandibular division (Figure 2B,F). By contrast, the expression of ATF3, which is a marker of nerve injury [42], was significantly increased in the ipsilateral TG at 3 days after IAMNT ( 2B,D,G). Compared to naïve animals, IAMNT operation increased the percentage of ATF3-IR neurons from 8.1 ± 2.2% to 54.3 ± 5.5%. There were no significant changes of ATF3 expression in sham-operated mice (17.1 ± 3.1%, *P* > 0.05 vs naïve, Figure 2B). ATF3-IR neurons were also restricted in the mandibular division of TG and no colocalized FG/ATF3 neurons were observed (Figure 2E,H).

### Upregulation of CCL2 in medullary astrocytes by IAMNT

**Figure 3:**
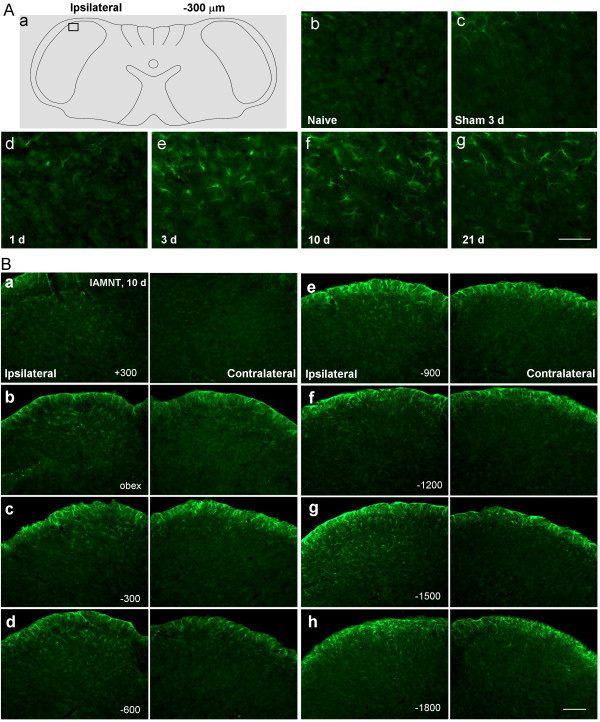
**The expression of CCL2 in the MDH following IAMNT. (A)** Temporal changes of CCL2-IR in the MDH following IAMNT at the level of 300 μm caudal to the obex. The area where the pictures were taken was shown in **(a)**. CCL2-IR was very mild in naïve mice **(b)**, but increased at 3 days **(e)**, 10 days **(f)**, and 21 days **(g)**. CCL2-IR was also mild in the sham-operated mice **(c)**. Scale bar, 50 μm. (**B)** Spatial changes of CCL2-IR in the MDH at 10 days after IAMNT. CCL2 expression was increased from 300 μm rostral to the obex **(a)** to 1500 μm caudal to the obex **(g)** in the ipsilateral side. However, no obvious change of CCL2 expression was found in the contralateral side of the MDH. Scale bar, 100 μm.

**Figure 4:**
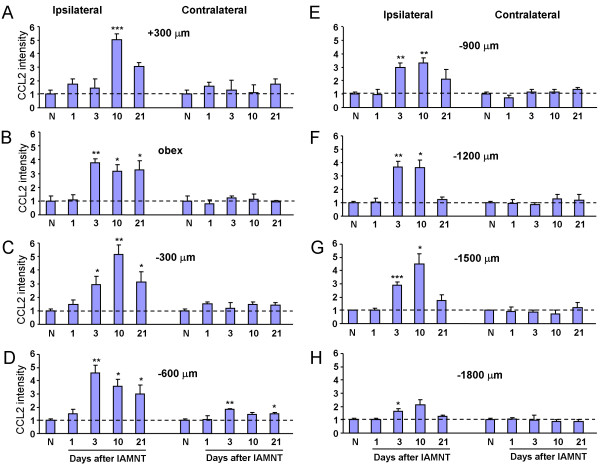
**Rostro-caudal (from + 300 μm to −1800 μm) distribution of CCL2-IR in both ipsilateral and contralateral MDH in naïve and IAMNT-operated animals (1 day, 3 days, 10 days, and 21 days).** * *P <* 0.05, ** *P <* 0.01, ***P < 0.001 compared to naïve. N, naïve. *n =* 6 for naïve, *n =* 5 for other groups.

We checked CCL2 expression from 1 day to 21 days after IAMNT. As illustrated in Figure 3, IAMNT increased CCL2 expression at day 3, day 10, and day 21 at the level of 300 μm caudal to the obex (Figure 3A). No significant increase of CCL2 expression was found in sham-operated animals (Figure 3Ac). CCL2 immunoreactive cells were mainly found in the superficial layers (Laminae I–III) and medial part of the dorsal horn. The representative rostro-caudal (from +300 μm to −1800 μm) photograph of CCL2 expression at 10 days after IAMNT was shown in Figure 3B. The statistical results of CCL2 expression from day 1 to day 21 at different levels were shown in Figure 4. Following IAMNT, CCL2 expression increased from day 3, peaked at day 10, and maintained at day 21. The increase of CCL2 expression was mainly in the ipsilateral MDH. However, at the level of −600 μm, CCL2 was also increased in the contralateral side at day 3 and day 21 (Figure 4).

**Figure 5:**
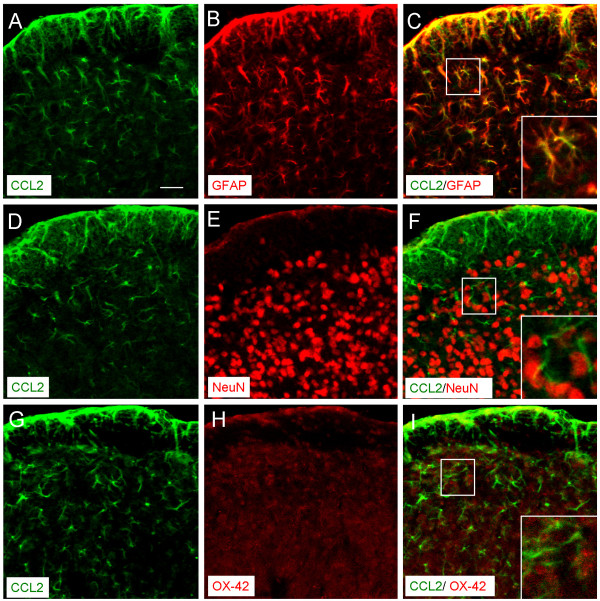
**Double staining of CCL2 with different cellular markers. CCL2 is mainly colocalized with astrocytic marker GFAP (A–C),but not with neuronal marker, NeuN(D–F),or microglial marker,OX-42(G–I).** Scale bar, 25 μm.

To define the cellular distribution of CCL2, we performed double staining of CCL2 with different cell markers. As shown in Figure 5, CCL2-IR was primarily colocalized with the astrocytic marker GFAP (Figure 5A–C), but not with neuronal marker NeuN (Figure 5D–F) or microglial marker OX-42 (Figure 5G–I), suggesting CCL2 is primarily induced in astrocytes in the MDH.

### Astrocytes activation in the MDH after IAMNT

**Figure 6:**
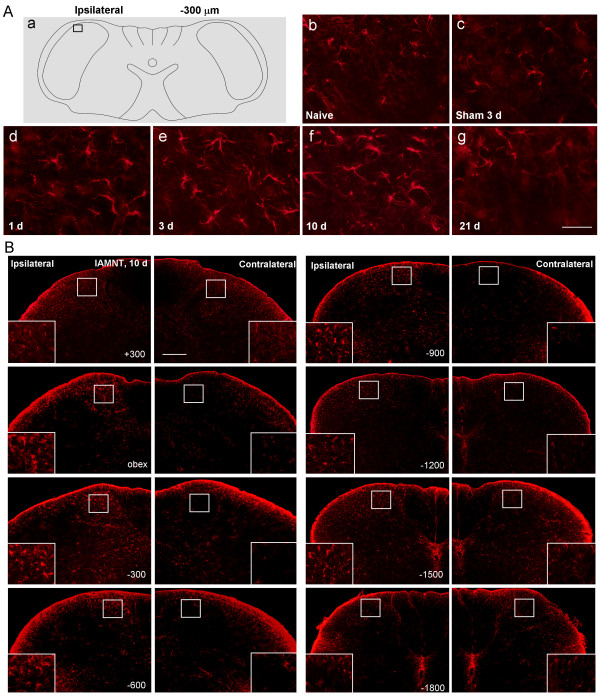
**The expression of GFAP in the MDH following IAMNT. (A)** Temporal changes of GFAP-IR in the MDH following IAMNT at the level of 300 μm caudal to obex. The area where the pictures were taken was shown in **(a)**. GFAP-IR was very mild in naïve mice **(b)**, but increased at 1 day **(d)**, 3 days **(e)**, 10 days **(f)**, and 21 days **(g)**. GFAP-IR was also mild in the sham-operated mice **(c)**. Scale bar, 50 μm. **(B)** Spatial changes of GFAP-IR in the MDH at 10 days after IAMNT. GFAP expression was increased from 300 μm rostral to the obex to 1800 μm caudal to the obex in the ipsilateral side. However, no obvious change of GFAP expression was found in the contralateral side of the MDH. Scale bar, 200 μm.

Because CCL2 is primarily expressed in astrocytes, we then examined astrocytes activation by checking GFAP expression in the MDH. In naïve and sham-operated animals, GFAP-positive astrocytes appeared to be in a resting state (Figure 6Ab,c). At 1 day after IAMNT, the astrocyte profiles appeared larger and had more processes compared to naïve (Figure 6Ad). Intense astrocytic responses were discernible in IAMNT animals on day 3 and day 10 (Figure 6Ae,f). We examined spatial changes of GFAP expression on day 10. As shown in Figure 6B, astrocytic activation was found from +300 to −1800 μm. The activated astrocytes were observed in the medial part of the superficial dorsal horn where intense CCL2-IR cells located.

### CCR2 upregulation in the MDH neurons after IAMNT

**Figure 7:**
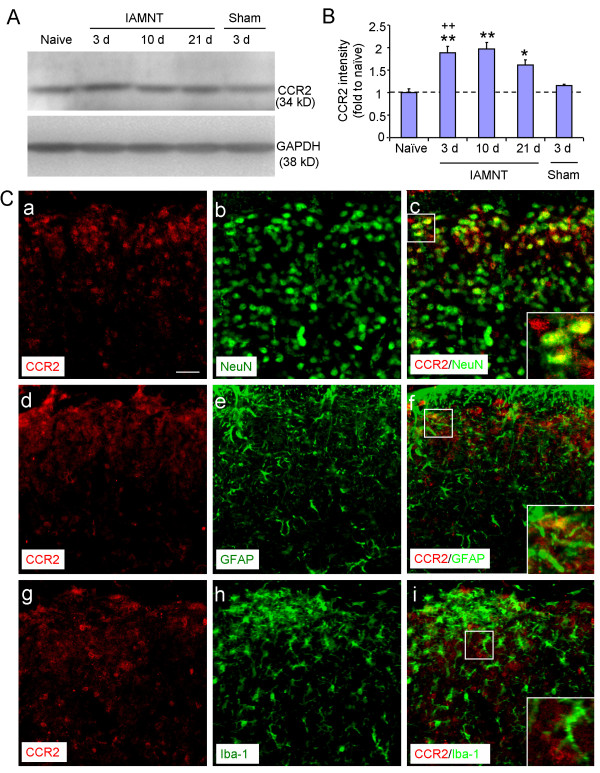
**The expression and cellular distribution of CCR2 in the MDH. (A)** A representative western blot shows CCR2 expression was increased in the ipsilateral medulla after IAMNT. **(B)** Density of CCR2 band, which is normalized to GAPDH loading control and expressed as ratio of naïve group. * *P <* 0.05, ** *P <* 0.01, compared to naïve. ++ *P* <0.01 compared to sham-operated group. *n =* 3 for each group. **(C)** Double staining of CCR2 with different cellular markers. CCR2 is colocalized with neuronal marker, NeuN **(a–c)**, astrocytic marker GFAP (**d–f)**, but not with microglial marker Iba-1 **(g–i)**. Scale bar, 25 μm.

CCR2 is a major type of receptor for CCL2 [43,44]. Western blot results showed that CCR2 expression in the ipsilateral medulla was significantly increased at 3, 10 and 21 days after IAMNT. Sham operated animals did not show obvious increase of CCR2 expression at 3 days (Figure 7A, B).

We then checked the cellular distribution of CCR2 by double staining of CCR2 with different cell markers. As shown in Figure 7C, CCR2-IR was primarily colocalized with the NeuN (Figure 7Ca–c). A few CCR2 positive cells in the superficial MDH were colocalized with GFAP (Figure 7Cd–f) and no cells were colocalized with microglial marker, Iba-1 (Figure 7Cg–i).

### Attenuation of IAMNT-induced pain hypersensitivity by CCR2 antagonist

**Figure 8:**
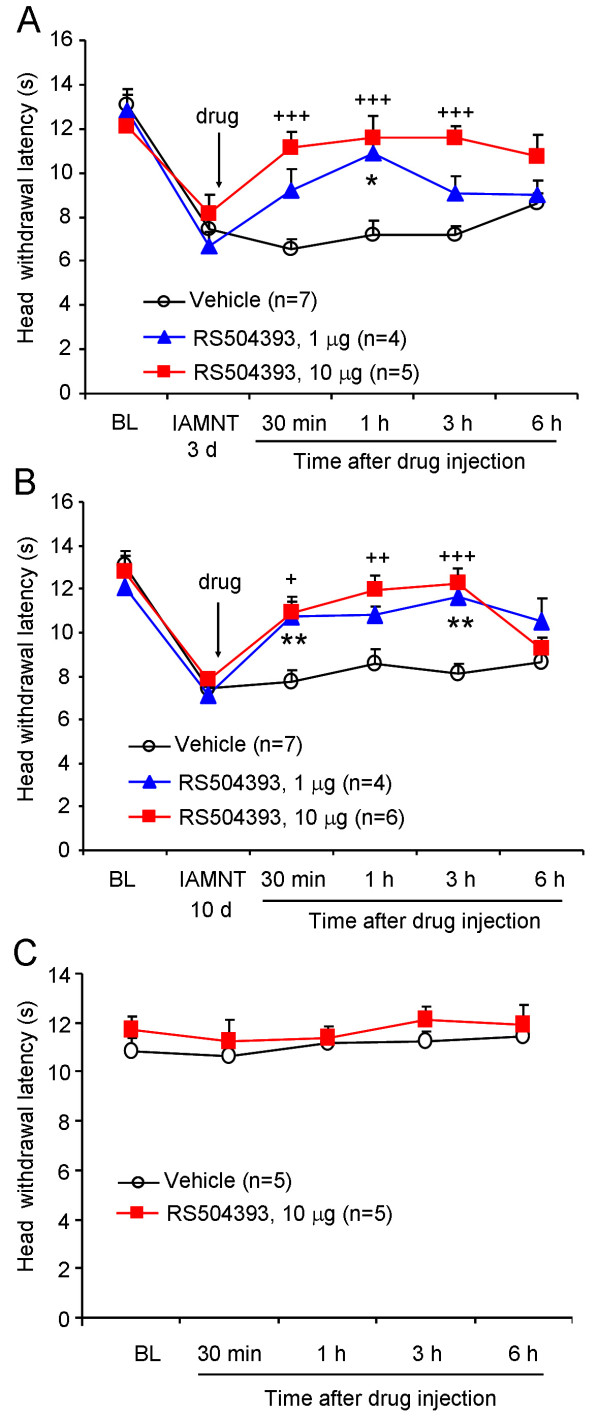
**The effect of RS504393 on IAMNT-induced heat hyperalgesia.** Intracisternal injection of RS504393, given at 3 days **(A)** or 10 days **(B)** after IAMNT, significantly reduced heat hyperalgesia on the ipsilateral facial skin. However, 10 μg RS50439310 did not affect the HWL in naive animals **(C)**. * *P <* 0.05, ** *P <* 0.01, 1 μg RS504393 versus vehicle; + *P <* 0.05, ++ *P <* 0.01, +++ *P <* 0.001, 10 μg RS504393 versus vehicle. BL, baseline.

As the significant upregulation of CCL2 and CCR2 was observed from day 3 after IAMNT, we speculate CCL2-CCR2 signaling may be involved the maintenance rather than the development of the trigeminal neuropathic pain. In addition, reversal of the established pain is more relevant than prevention in clinic. Therefore, we used an extremely selective CCR2 chemokine receptor antagonist, RS504393 and tested its analgesic effect by intracisternal injection at 3 days or 10 days after IAMNT. As shown in Figure 8A, the IAMNT significantly decreased HWL in the ipsilateral side at 3 days after the injury. The vehicle did not affect the IAMNT-induced heat hyperalgesia, whereas intracisternal injection of 1 μg of RS504393 significantly increased the HWL at 1 hour (*P <* 0.05). RS504393 at the dose of 10 μg showed stronger effect than that of 1 μg and the effect started from 30 minutes, maintained at 3 hours and recovered at 6 hours (Figure 8A). Similarly, intracisternal injection of 1 μg or 10 μg of RS504393 also blocked the heat hyperalgesia at 10 days after IAMNT (Figure 8B). The effect of RS504393 was also maintained for more than 3 hours. 

To test the effect of RS504393 on basal nociception, we intracisternally injected 10 μg RS504393 in naïve animals and tested the HWL. As shown in Figure 8C, RS405393 at the dose of 10 μg did not affect the HWL from 30 minutes to 6 hours. The results indicate that CCR2 may not be involved in the trigeminal nociceptive pathway in normal conditions.

To evaluate whether antinociceptive doses of CCR2 antagonist are associated with motor dysfunction, a rotarod test was performed following intracisternal injection of RS504393 (1, 10 μg). Neither 1 μg nor 10 μg affected the motor performance at 30 minutes, 1 hour, and 3 hours after RS504393 administration (data not shown).

## Discussion

In this study, we characterized the morphological and functional profile of CCL2 and CCR2 in the MDH in response to peripheral nerve injury. Our results demonstrated first that IAMNT induced persistent heat hyperalgesia that was associated with ATF3 expression in the TG and persistent CCL2 upregulation in the MDH astrocytes. Second, CCR2, the major receptor of CCL2 was increased from 3 days to 21 days after IAMNT and CCR2 was mainly expressed in neurons. Third, intracisternal injection of CCR2 antagonist attenuated IAMNT-induced heat hyperalgesia at 3 days and 10 days after IAMNT. These data suggest that CCL2-CCR2 signaling in the MDH play an important role in the maintenance of orofacial neuropathic pain.

ATF3 is a member of the activating transcription factor/cAMP-responsive element binding protein (ATF/CREB) family and has been widely used as a neuronal marker of nerve injury in the nervous system [42]. In this study, we observed that ATF3 expression was robustly increased in the ipsilateral side of mandibular division of TG, whereas the FG-labeled neurons were significantly decreased at 3 days after IAMNT. Although ATF3 was not expressed in the maxillary division of TG, the non-ATF3-expressing neurons may contribute to the heat hyperalgesia in the whisker pad area after IAMNT transection. Tsuboi *et al*. reported that after transection of the IAN, the A-fiber activities of the ION were significantly enhanced and the excitability of TG neurons innervated by the ION was increased [40]. In addition, Tsuzuki *et al*. showed that preprotachykinin mRNA was increased in the uninjured mandibular neurons after ION transection [45], suggesting that the intact neurons may contribute to the secondary hyperalgesia at the ganglion level. However, whether CCL2-CCR2 signaling in the TG is involved in the secondary hyperalgesia needs to be investigated in the future.

Previous studies have reported that CCL2 is constitutively expressed in small and medium neurons of the DRG and the expression is upregulated after nerve injury [28,46]. In the spinal cord, CCL2 expression is also upregulated under neuropathic conditions [29,46,47]. The present results showed that CCL2 expression was significantly increased in the MDH during day 3 to day 21 from the level of obex to −1800 μm caudal to the obex. The lamina distribution showed that CCL2 was mainly expressed in laminae I–III of the medial part of the dorsal horn. Double staining further showed CCL2 was predominantly induced in astrocytes after IAMNT, which is consistent with the following reports. First, the CCL2 upregulation is found in spinal astrocytes after spinal nerve ligation [47] and spinal cord contusion injuries [48]. Second, CCL2 is expressed in brain astrocytes in pathological conditions, such as experimental autoimmune encephalomyelitis [49-51], brain ischemia [52,53], traumatic brain damage [54], and Alzheimer’s disease [55]. Third, *in vitro* studies show that primary cultured astrocytes produce CCL2 [47,56-58]. Finally, mice overexpressing CCL2 in astrocytes display enhanced nociceptive responses [26]. However, we do not exclude the possibility that IAMNT could induce CCL2 release from the central terminals of primary afferents, as previous studies showed that CCL2 is expressed in CGRP-immunoreactive primary afferents in the spinal cord and increased under neuropathic conditions [29,46].

It is becoming clear that astrocytes and microglia are activated after peripheral nerve injury or inflammation [59-61], and glial activation might be a causal factor in the pain hypersensitivity at the spinal level. Similarly, glial activation was also induced in the MDH after trigeminal nerve ligation or transection [9-11,36], orofacial inflammation [11,62], and tumor cells inoculation in the vibrissal pad [63]. Consistent with these studies, we found that the astrocytes in the MDH changed their morphological features and manifested large somata with many thick processes from 3 days to 21 days after IAMNT. In addition, the astrocytic activation was correlated with the heat hyperalgesia behavior and CCL2 upregulation. Okada-Ogawa *et al*. have reported that medullary application of the astroglial metabolic inhibitor sodium fluoroacetate attenuated heat hyperalgesia at 7 days after the IAN transection in rats [36], supporting the view that medullary astrocytes are involved in the maintenance of orofacial neuropathic pain.

Astrocytes are the most abundant cells and have a close contact with neurons and blood vessels. They not only support and nourish neurons but also regulate nearby neuronal excitability and glial functions by the release of gliotransmitters such as ATP, glutamate, growth factors (BDNF, bFGF), proinflammatory cytokines (e.g. IL-1β, TNF-α), and chemokines (CCL2, CXCL1, CXCL10) [47,62,64,65]. Glial–neuronal interaction has been implicated to contribute to central sensitization under pathological conditions [32]. For example, IL-1β is a major proinflammatory cytokine and upregulated in astrocytes in chronic pain conditions [62,66,67] . Immunostaining shows that IL-1 receptor type 1 colocalizes with the N-Methyl-D-aspartate (NMDA) receptor NR1 subunits in neurons of the spinal cord [68], trigeminal nucleus [62], and rostral ventromedial medulla [67]. Importantly, IL-1β can directly modulate synaptic transmission in the spinal cord by enhancing excitatory synaptic transmission and suppressing inhibitory synaptic transmission [69]. Here, CCL2 was clearly shown in medullary astrocytes, and its preferred receptor, CCR2 was mainly expressed in neurons, indicating that CCL2-CCR2 might be involved in the astroglial–neuronal signaling in the MDH.

Although our immunostaining results showed the predominant expression of CCR2 in neurons, the cellular localization of CCR2 in the central nervous system is heavily debated. An early immunohistochemical study shows CCR2 expression in spinal microglia and spinal administration of CCL2 activates microglia [24,25]. However, the presence of CCR2 in microglia has recently been questioned by the work of Saederup, demonstrating a CCR2 expressing population of cells did not express CX3CR1, which is known to be expressed exclusively in microglia [70]. This suggests microglia might not express CCR2, and CCR2 positive cells are perhaps infiltrating monocytes [33,70]. The expression of CCR2 in neurons can be supported by recent evidence. It has been reported that CCR2–green fluorescent protein (GFP) reporter mice [27] show a weak but clear GFP signal in dorsal horn neurons [47]. *In situ* hybridization studies showed that CCR2 mRNA was increased in dorsal horn neurons after spinal nerve ligation [47]. Spinal administration of CCL2 induces extracellular signal-regulated kinase activation in the spinal cord neurons [47,71]. Furthermore, CCL2 rapidly increases spontaneous excitatory postsynaptic currents and NMDA-induced current [47] and inhibits GABA-induced currents [72] in dorsal horn neurons. CCR2 has also been shown to be upregulated in the spinal astrocytes following spinal cord injury [48] and hippocampal astrocytes after status epilepticus [73]. In agreement with these results, our results showed few CCR2-IR cells were also observed in astrocytes in the superficial dorsal horn.

It has been reported that CCR2 is increased in the DRG or spinal cord following partial sciatic nerve injury or spinal nerve ligation [24,47]. The present study further showed a persistent increase of CCR2 protein in the ipsilateral medulla after IAMNT. In parallel with these results, behavioral data showed that inhibition of CCR2 by intracisternal injection of RS504393 attenuated IAMNT-induced heat hyperalgesia of the whisker pad area at both 3 days and 10 days after the surgery, indicating that CCR2 in the MDH contribute to the secondary hyperalgesia. The previous results showed that the use of CCR2 receptor antagonists or blocking antibodies successfully inhibited nociceptive signaling [71,74-76]. In addition, mice lacking CCR2 display reduced mechanical allodynia after partial ligation of the sciatic nerve [24,25]. These data indicate that CCR2 play a key role in the maintenance of neuropathic pain at both the spinal and trigeminal level.

## Conclusions

In this study, we demonstrated that CCL2 and CCR2 were persistently increased in the MDH following IAMNT with the CCL2 expression in activated astrocytes and CCR2 expression in neurons. Inhibition of CCR2 by selective CCR2 antagonist attenuated IAMNT-induced secondary heat hyperalgesia. These data indicate that CCL2 and CCR2 in the trigeminal spinal subnucleus caudalis can serve as signaling molecules between astrocytes and neurons and contribute to the maintenance of neuropathic pain. Targeting the CCL2/CCR2 pathway may provide a novel therapeutic approach for the treatment of the trigeminal neuralgia.

## Abbreviations

BDNF: brain-derived neurotrophic factor; bFGF: basic fibroblast growth factor; CCL2: chemokine C-C motif ligand 2; CCR2: chemokine C-C motif receptor 2; DRG: dorsal root ganglion; ECL: enhanced chemiluminescence; GAPDH: glyceraldehyde-3-phosphate dehydrogenase; GFAP: glial fibrillary acidic protein; GFP: green fluorescent protein; HWL: head-withdrawal latency; IAMN: inferior alveolar nerve and mental nerve; IAMNT: inferior alveolar nerve and mental nerve transection; IAN: inferior alveolar nerve; IL-1β: interleukin-1β; ION: infraorbital nerve; MAPK: mitogen-activated protein kinase; MCP-1: monocyte chemoattractant protein-1; MDH: medullary dorsal horn; MN: mental nerve; NMDA: N-Methyl-D-aspartate; PBS: phosphate-buffered saline; TNF-α: tumor necrosis factor-α; TG: trigeminal ganglion.

## Competing interests

The authors declare that they have no competing interests.

## Authors’ contributions

ZJZ carried out the animal surgery, behavioral testing, immunohistochemistry and western blot experiments. YLD participated in behavioral testing. YL and SC participated in data analysis. ZQZ contributed to the preparation of the manuscript. YJG conceived of the project, coordinated and supervised the experiments, and wrote the manuscript. All authors read and approved the final manuscript.
